# The promising use of nano-molecular imprinted templates for improved SARS-CoV-2 detection, drug delivery and research

**DOI:** 10.1186/s12951-021-01032-x

**Published:** 2021-10-06

**Authors:** Alaa F. Nahhas, Thomas J. Webster

**Affiliations:** 1 Biochemistry Department, College of Science, King Abdulaziz University, Jeddah, 21589 Saudi Arabia; 2 Department of Chemical Engineering, College of Engineering, Northeastern University, Boston, MA 02115 United States

**Keywords:** Molecular imprinted polymers, Plastic antibody, Artificial cell receptors, Nanocarriers, COVID-19

## Abstract

Molecular imprinting (MI) is a technique that creates a template of a molecule for improving complementary binding sites in terms of size and shape to a peptide, protein, bacteria, mammalian cell, or virus on soft materials (such as polymers, hydrogels, or self-assembled materials). MI has been widely investigated for over 90 years in various industries but is now focused on improved tissue engineering, regenerative medicine, drug delivery, sensors, diagnostics, therapeutics and other medical applications. Molecular targets that have been studied so far in MI include those for the major antigenic determinants of microorganisms (like bacteria or viruses) leading to innovations in disease diagnosis via solid-phase extraction separation and biomimetic sensors. As such, although not widely investigated yet, MI demonstrates much promise for improving the detection of and treatment for the current Coronavirus Disease of 2019 (COVID-2019) pandemic as well as future pandemics. In this manner, this review will introduce the numerous applications of MI polymers, particularly using proteins and peptides, and how these MI polymers can be used as improved diagnostic and therapeutic tools for COVID-19.

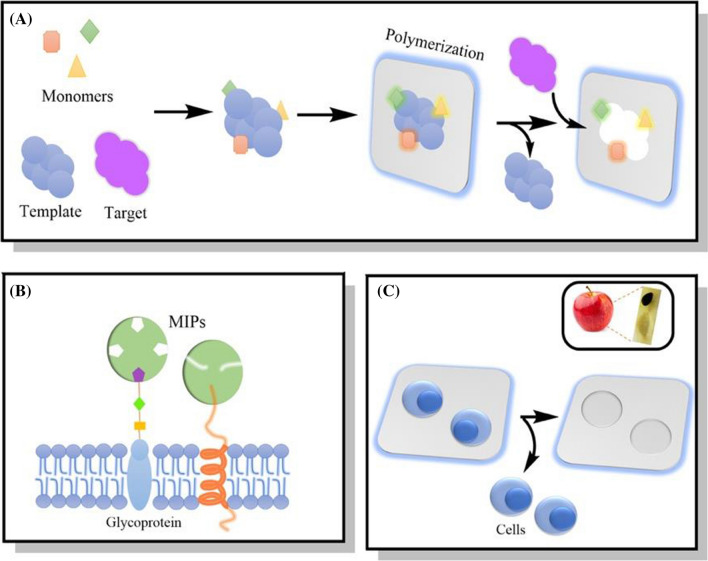

## Introduction

### Fundamentals of molecular imprinting (MI)

**Fig. 1:**
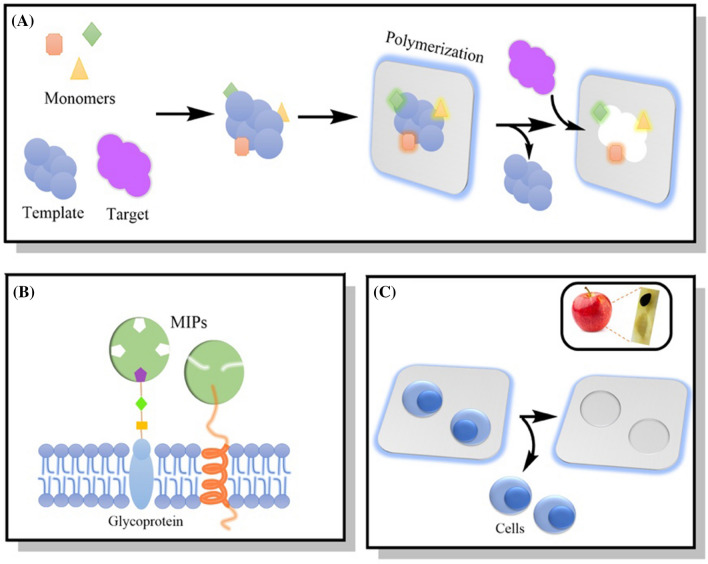
**A** A representative example of the molecular imprinting process; **B** MIPs as recognition sites for cell-based molecules; and **C** MIPs using an entire cell. Upper right: an example from a natural occurring MI process that shows an apple seed-hole acting as a templet for the seed

Molecular imprinted polymers (MIPs) are created through a process that can solve problems associated with using a specific living system by creating artificial antibodies, receptors, or even a specific aptamer as a recognition site for binding to a virus, bacteria, mammalian cell, or any other biomolecule. This method can be used as a template to design a specific targeting sequence in bacteria or viruses using synthetic polymers that are stabilized by covalent, non-covalent, ionic, semi-covalent, or metal center coordination binding interactions.[1, 2] The template can then be removed from a polymerizable group leaving behind an empty cavity that is called imprinting as shown in Fig. 1A [3].

As just one of many examples, for the improved detection of bacteria in a solution, the surface of *Staphylococcus aureus* (SpA) which contains a specific protein A, can be imprinted on a polymer, removed, and then used to improve SpA attachment for detection. In fact, Xue and his co-workers imprinted protein A on polyacrylamide gel beads then removed the protein leaving behind an empty cavity on the beads showing high selectivity toward SpA with an adsorption capacity of 10^3^-10^4^ CFU; other bacteria did not show any binding to the cavity thus demonstrating high specificity for SpA. Such results, and more, illustrate how MIPs improve material-target specificity without complicated chemistry, surface functionalization strategies, and expense [4].

In yet another example, entire bacteria (like *Escherichia coli (E. coil)*) can also be imprinted on devices to improve their sensitivity for detection in water. Yilmaz and his team imprinted whole cells on sensitive devices to detect the presence of bacteria in water and used N-methacryloyl-L-histidine methylester, which is a polymer of histidine, as a recognition element for natural antibodies [5].

### MI methods

The MI method for microorganisms can be classified into direct and indirect methods. Direct imprinting involves film imprinting which is easy to use and suitable for fragile samples, however, it needs a large sample [6]. The indirect imprint method is used for artificial templates where the targeting template is very hard to deal with or unavailable, like a virus or unstable molecule which is clearly not easy to handle, needs contact avoidance, and needs numerous imprinting steps further complicating the direct imprinting process [7]. In the indirect method, we cannot use the polymer to be imprinted with a targeting analyte itself, like Coronavirus, but we can use it with other molecules that give the same selectivity. One way to use the indirect method is by creating artificial template stamps. The first step is imprinting the natural template on a polymer then washing it to leave the empty cavity for the second prepolymer. The second polymer leads to the artificial template and that can be used to create a sensing surface to be used several times [1, 8].

When designing MIPs for specific recognition purposes, two methods must be considered: targeting a specific cell-membrane molecule (such as proteins, lipids, or glycans) or un-specifically targeting the whole cell or microorganism as again shown in Fig. 1 [9, 10]. Table 1 summarizes some of the more impressive MIP binding approaches to specific molecules on cell membranes and their biological functions.

**Table 1:** Molecular imprinted methods and their applications in bioimaging

Template	Monomer	Crosslinker	Applications
Sialic acid (SA)-imprinted conjugated polymer nanoprobe	Poly(fluorene-alt-benzothiadiazole)- Phenylboronic acid (PFBT-PBA)	None	Targeting sialylated glycan levels using 2-photon fluorescent imaging [11] and cancer imaging [10]
Glucuronic acid (GlcA)	4-Acetoxy-4′-acetylbiphenyl (AAB), methacrylamide (MAM)	Ethylene glycol dimethacrylate (EGDMA)	Imaging hyaluronan on cells and skin tissue [12] or targeting hyaluronan in HeLa cells [13]
*N*-Acetylneuraminic acid (NANA) and GlcA	MAM, 4′-acetylbiphenyl (AB)	EGDM	Imaging and labeling of hyaluronan and sialic acid [14]
l-Phenylalanin-amide	Methacrylic acid glacial (MAA)	EGDM	Studying the binding affinity between ligands and receptors [15]
Epidermal growth factor receptor (EGFR)	*N*-Isopropylacrylamide (NIPAAm)	None	Targeting living cells [16]
SA, Fucose (Fuc), Mannose (Man)	Fluorescent polymerization-based amplification (FPBA), tetraethoxysilane (TEOS)		Targeting and imaging of cancer cells [17]

### MI with small/big molecules like peptides or proteins

There are many advantages towards using polymers in MIs, such as their improved specific binding, stability, inexpensive nature, delivery, wide use, biosensing capability, and the ability to use artificial entities (like antibodies, proteins, viruses, bacteria, etc.) compared to hazardous entities [18]. The imprinting process can use cell membrane receptors, entire cells, proteins, peptides, and so many more difficult to use biological agents [19]. Peptides and proteins have been shown to be more useful in MIPs than other biological molecules (like lipids or glycans) due to their more stable properties advantageous during the MI process to overcome high or low temperatures, high salt media, various structures, and a wide range of pH values. For example, the entire fibronectin (FN) protein was used as a template and was imprinted on a polysiloxane membrane to enhance mouse fibroblast cell (L929) growth and cell adhesion than non-imprinting polymers since FN is a protein that promotes cell proliferation and helps in cell adhesion [20].

Such MIP processes are encouraging since cell membrane proteins are very expensive for such use and can lead to regulatory issues. Researchers have instead used small peptide sequences or epitopes in MI and have outlined a specific strategy to use such select epitopes. For example, specific target sequences of these peptides can be selected from https://www.uniprot.org/. Examples of such epitope imprinting peptides include imprinting arginylglycylaspartic acid (RGD), which is widely known to improve cell adhesion, for tissue engineering applications [21]. In addition to these peptides, glycomoieties on the cell surface have been widely imprinted, especially mannose and sialic acid (SA) [2]. SA is usually overexpressed on cancer cells and it can be used as a biomarker to detect the presence of metastasis in the body by using MIP to detect it [2].

In addition, Drzazgowska and his coworkers designed a novel epitope peptide containing a double cysteine as a template using an imprinting procedure and adsorbed these peptides onto a gold surface after allowing them to form a self-assembled monolayer (SAM) bridge [22]. The sequence of this peptide was CKGVLKAVDHINSTIAPC which was computationally selected from the neuron-specific enolase (NSE) [23]. NSE is a biomarker that is overexpressed on small cell lung cancer cells [24]. The polymer network was cured using electro-polymerization to molecularly imprint the peptides. The cavities after the washing step were stabilized by hydrophobic interactions and hydrogen bonds to capture the target protein during the rebinding step. The surface exhibited a high affinity toward a target protein serving as a much more effective cancer biomarker with a twelve-time lower concentration than that of a traditional biomarker and higher specificity compared to traditional materials, such as those vertically adsorbed [22]. From these promising results, it is clear that MIPs could be used as a sensor for detecting numerous biomarkers.

Sugar receptors (like fucose or mannose) have been used for targeting cancer cells because such sugars are overexpressed on the surface of these cells (26, 27). Monitoring such sugar or even sialylated glycans in cells can be used as a diagnostic tool or even for the treatment of diseases like cancer. For example, Liu and his group imprinted monosaccharides with fluorescent nanoparticles to image cancer cells and found that this method specifically targeted human hepatoma carcinoma cells (HepG-2) over normal mammary epithelial cells (MCF-10 A) [17]. This is a promising result to be used as a probe for targeting cancer cells. Targeting an entire virus or bacteria membrane could be very useful for detecting harmful viruses in a solution that may cause an infection. Targeting the whole cell is further stabilized by cell-shape complementary mechanisms (i.e., lock and key mechanisms) and non-covalent interactions between the MIP matrices and the cell membrane.

**Fig. 2:**
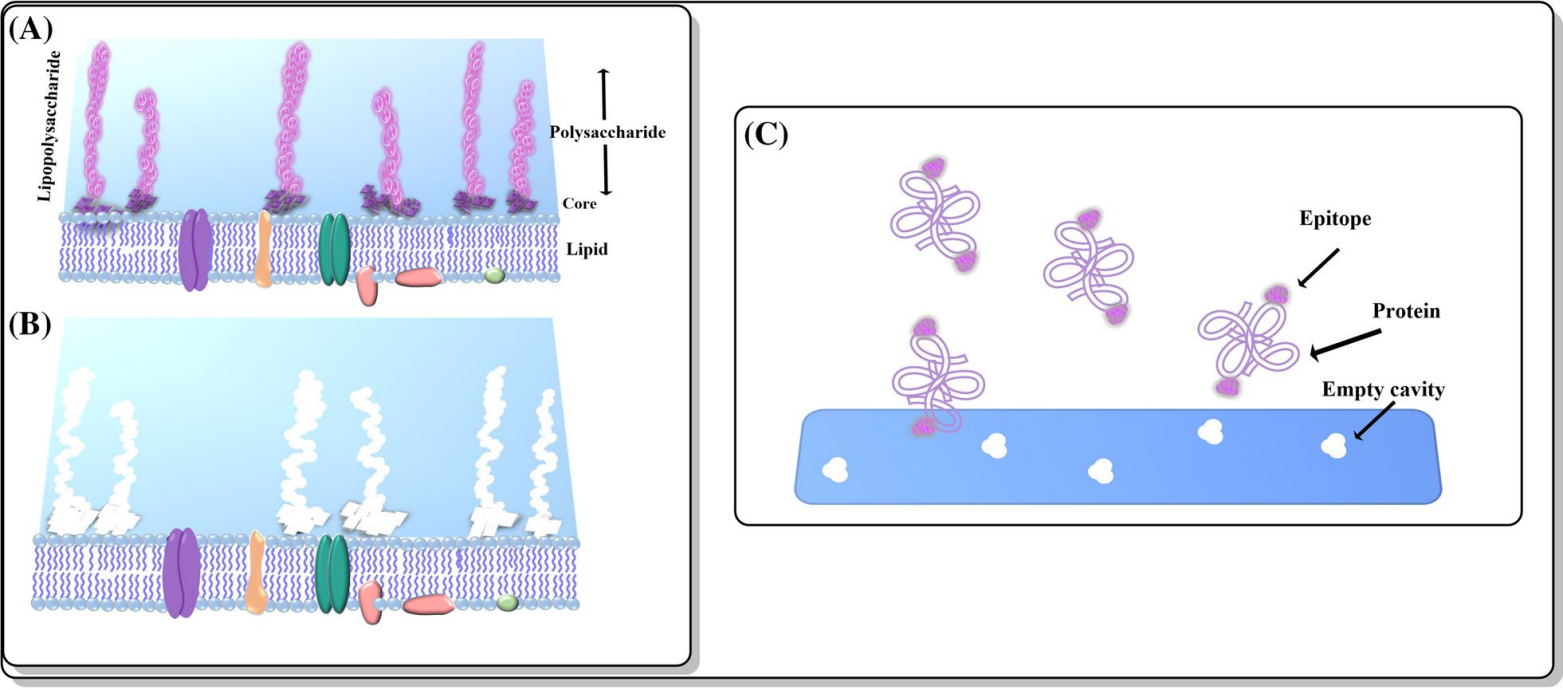
Different MI templates using saccharides and peptides as epitopes. **A** Outer cell membrane containing polysaccharides as glycans; **B** The outer membrane template after washing the MIP; **C** The fibronectin imprinted material enhanced cell adhesion after 24 h using Arg-Gly-Asp-Ser

Likewise, glycans from different types of cancer cells have been distinguished by using MIPs [2]. Figure 2 shows a MI approach using peptides and saccharides. However, sometimes imprinted proteins denature and lose their shape or cannot be removed after the washing step to leave an empty cavity due to harsh conditions [27].

This review paper will cover numerous applications of MIPs, particularly those imprinting protein and peptides, as improved diagnostic tools for the early detection of pathogens that are urgently needed due to the recent COVID-19 pandemic and ongoing worldwide cancer problems.

### Fundamentals of MI small molecules

#### Artificial cell receptors

**Fig. 3:**
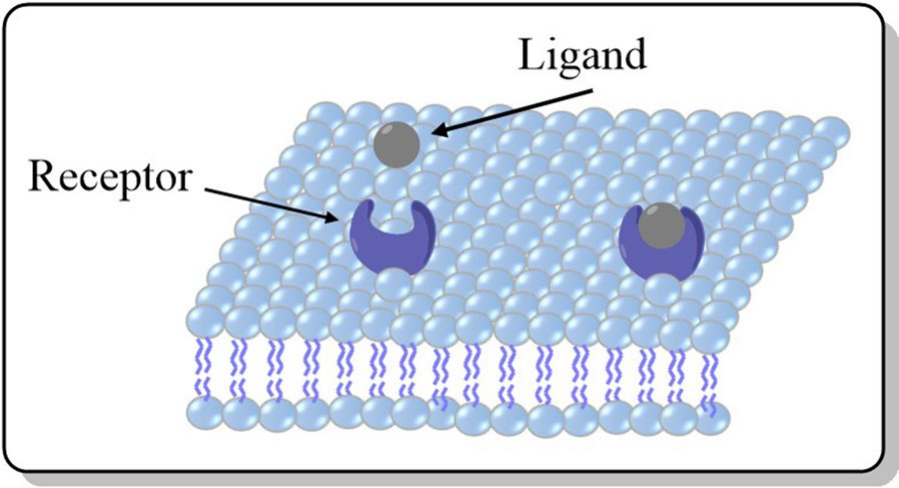
A representative example of the complementary structure formed between a natural receptor and ligand complex

MIPs have been used to synthesize what are called “artificial receptors” that look like real cell receptors and exhibit properties similar to a human cell or microorganism receptor. Figure 3 shows the natural recognition between a cell receptor and ligand. Attractive properties of the artificial receptors include their increased stability, often times they are safer to work with making them potential tools in biomedicine.

**Fig. 4:**
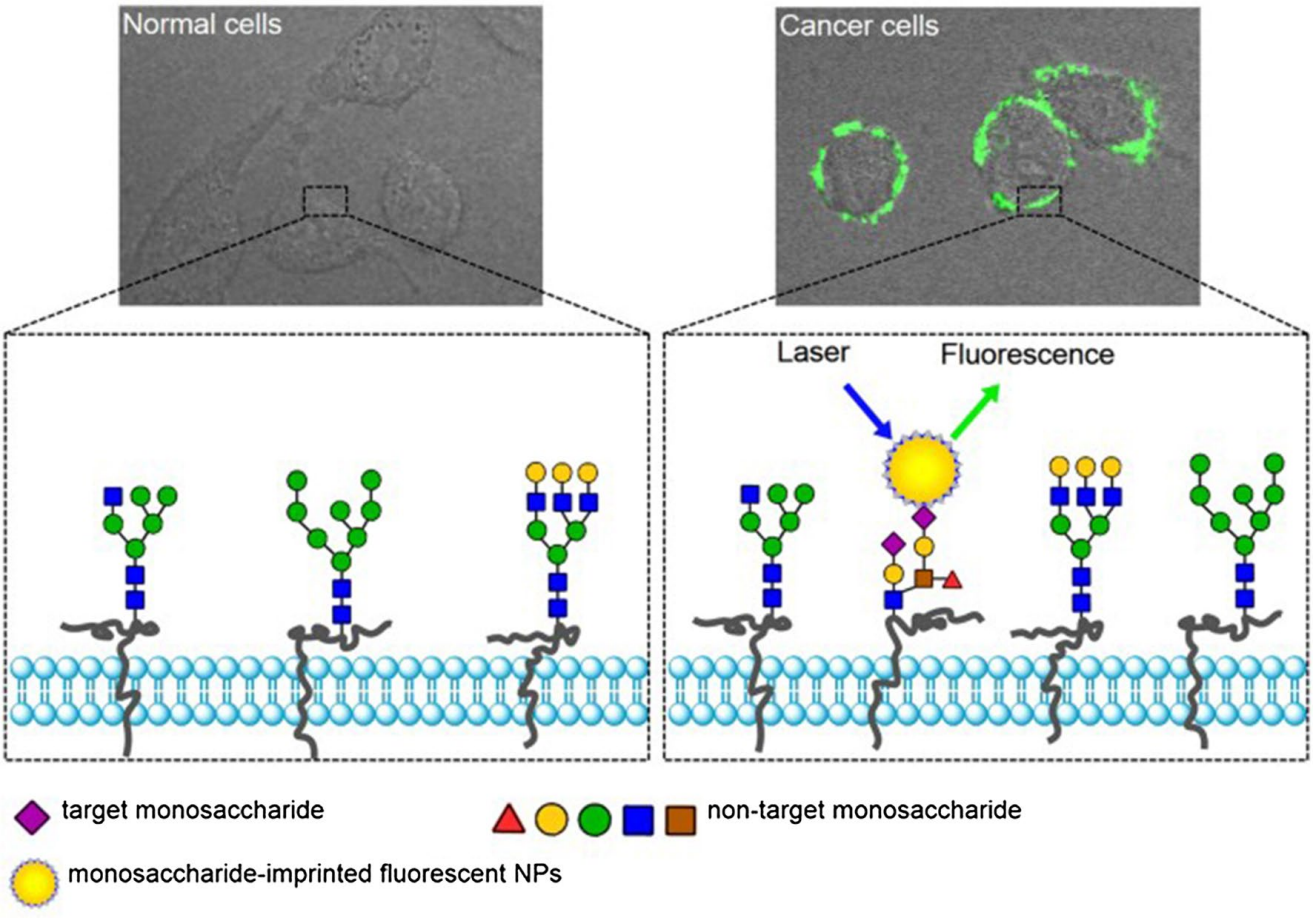
Monosaccharide (sialic acid)- imprinted fluorescent nanoparticle used as a bioimaging indicator to designate normal versus cancer cells. This MIP probe consists of fluorescein isothiocyanate (FITC) doped silca nanoparticles imprinted with monosaccharide as a template. Adapted from [17]

There are extensive studies relating such materials to MI since these molecules bind to their ligands in a typical biological lock and key mechanism [18]. Artificial receptors are able to bind and interact with targeted cells and change their functions, which needs to be considered when designing such receptors. Changing the target cell function is the main consideration that researchers take into account when designing receptor-like cell membranes. MIPs can then help in cell differentiation [29], cell apoptosis [30], or cell adhesion [21]. In addition to all of these applications, MIPs can help in cell recognition attracting huge attention in the biomedical field. For example, imprinting of Epidermal Growth Factor Receptor (EGFR) epitopes (which are overexpressed in cancer cells) can be accomplished using *N*-isopropylacrylamide (NIPAm) based molecules [31]. These MIPs were synthesized on glass beads with a peptide template using a solid-phase method followed by washing the low-affinity monomers and polymers with low temperatures, then by raising the temperature to 60 °C to remove high-affinity binding monomers and polymers. The function of these MIPs is to distinguish between different levels of growth factor expression between cancer cells [31]. Glycans on cancer cells, such as fucose and mannose, were also used as targets for synthetic receptors. These glycans were imprinted on silica nanoparticles with boronate. These nanoparticles fluoresce for use in cancer cell imaging and to differentiate between cancer and healthy tissue where overexpression of these glycans occurs compared to normal cells as shown in Fig. 4 [17]. Also, a thiolated DNA aptamer and MIP hybridized into a synthetic sensor receptor onto a gold surface electrode was developed for electrochemical detection of a prostate-specific antigen [32].

### Plastic antibody

**Fig. 5:**
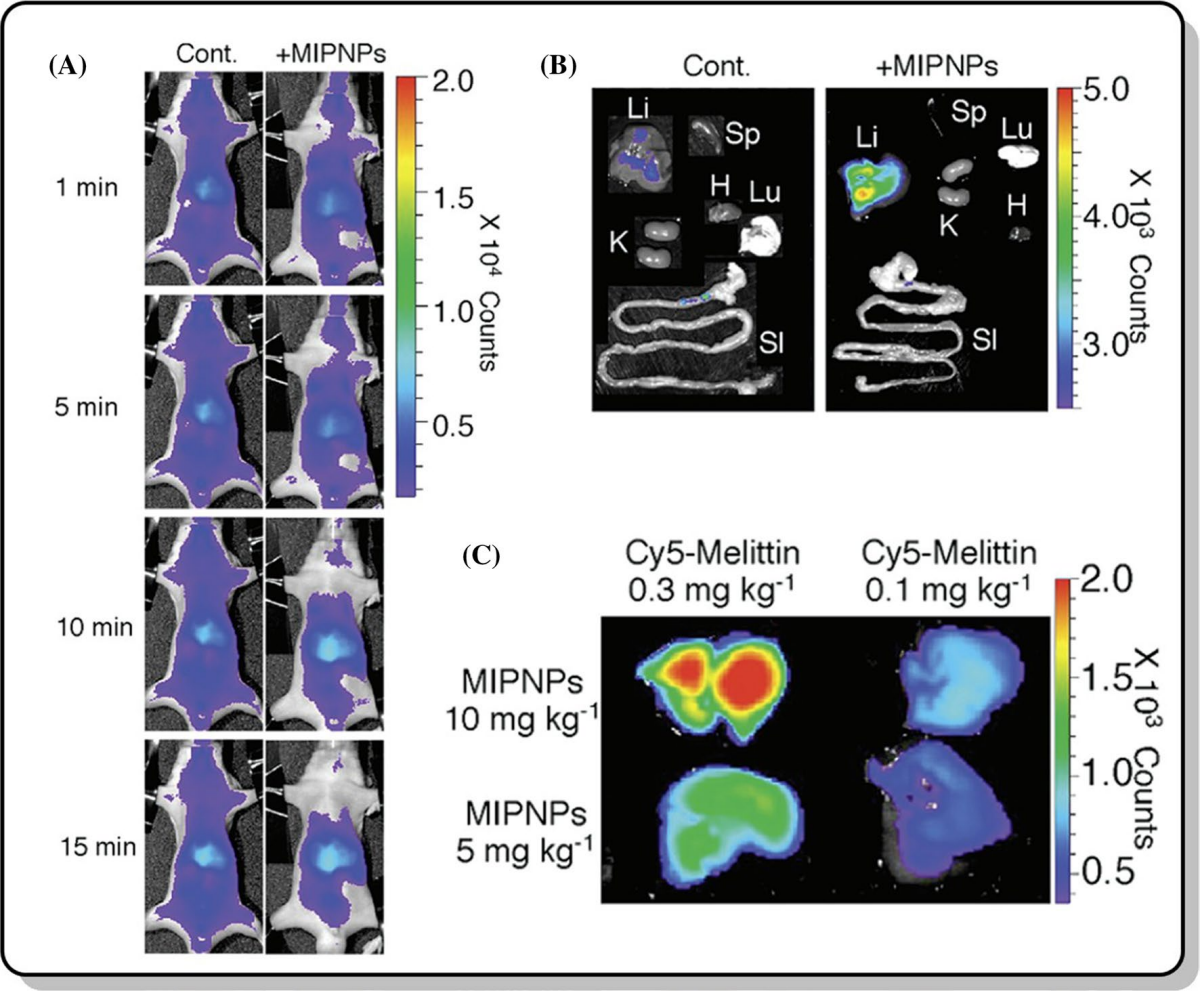
**a** Biodistribution of the toxic peptide (1 mg kg^− 1^) and 27 mg kg^− 1^ of the MIP during different time frames; **b** Fluorescent tracking of the toxic peptide and MIP in mice livers after 70 min of injection; and **c** Distribution of the toxic peptide and MIP in the different organs of the mice. Adapted from [34]

Plastic or synthetic antibodies use the same idea as artificial cell receptors and possess many of the same advantages as the MI structures discussed. They have many advantages over natural antibodies due to their stability, safety, inexpensiveness, reusability, lack of needing animals for antibody production, resistance to being degraded by proteolytic enzymes, and ease of synthesis [18, 33]. Specifically, Shea and his coworkers designed imprinted polymers based on the toxic peptide melittin (GIGAVLKVLTTGLPALISWIKRKRQQ) present in bee venom to create a synthetic antibody against that peptide [34]. They found that the survival time when injecting MIP into mice was longer than without using it [34]. They also found that melittin-imprinted nanoparticles were non-toxic in vitro at concentrations ranging from 3 to 3000 µg/ml [34]. From these results, MIPs have numerous biomedical applications by capturing a toxic peptide (such as melittin) and allowing it enter into the bloodstream without any toxicity. Figure 5 shows the biodistribution of the toxic peptides and the MIPs used in that study.

### MI viruses

#### MIPs to improve virus detection and therapies

The Coronavirus Disease of 2019 (COVID-2019) is a recent pandemic which has stopped the world, resulting in numerous deaths, and continues to spread to this day with different mutations. Many researchers have focused on ways to decrease SARS-CoV-2 (the virus causing COVID-19) exposure, increase detection and enhance passivation therapies since 2019. Unfortunately, SARS-CoV-2 research needs to be completed in a biosafety III level laboratory due to the highly contagious nature of SARS-CoV-2, which has significantly hampered progress towards treating this pandemic. Clearly, there has been and continues to be an urgent need to find other research tools to diagnose and detect such dangerous viruses as SARS-CoV-2.

Using specific living systems (such as bacteria or viruses) for the creation of advanced medical materials and/or advanced medical research, however, can be difficult and dangerous to our health, especially if they cause severe diseases, such as the recent COVID-19. As just one of many examples, advances in the prevention, diagnosis, and treatment of COVID-19 could have undoubtedly been expedited if research groups around the world could use MI materials to mimic SARS-CoV-2 rather than using the actual virus, which in most cases would not have been possible anyway. The most common way for the early detection of such viral diseases is by reverse transcription-polymerase chain reaction (RT-PCR) [35–37], however, RT-PCR uses a significant amount of time in order to detect the presence or absence of the virus and needs skilled personnel with specialized equipment. Also, the enzyme-linked immunosorbent assay (ELISA) with a double antibody sandwich assay can be used to detect viruses in the patient’s serum of nucleocapsid protein which is one of the four proteins in the virus in its early stages [38]. However, this is not a reliable method since antibodies appear after ~ 14 days [39]. There are many other diagnostic tools but they all depend on the presence of antibodies, such as the test that depends on a fluorescence immunochromatographic assay [40], and a field-effect transistor-based biosensing device [41]. Chest Computed Tomography (CT) is also another way to detect virus infection sites in the lung [42], but this diagnosis needs an expert to detect viruses and it is expensive [43]. Clearly, this current COVID-19 pandemic has highlighted a need for new materials that can mimic viruses for improved virus research, medical products, and rapid detecting tests-something MI can provide.

#### MIPs to improve virus research including Coronavirus

MIPs use templates to improve the binding affinity between a cell or virus receptors and ligands depending on the particle’s size or material shape. Specifically, Cumbo and his coworkers designed nanoparticles that imprint a virus structure on its surface and termed such structures as virus imprinted polymers (VIPs) [44]. Their strategy was based on designing the surface of empty cavities that remain after polymer deposition and template removal. They imprinted the small RNA plant virus, such as *Turnip yellow mosaic virus* and *tomato bushy stunt virus*, on the surface of silica nanoparticles (SNPs). Basically, this strategy is divided into three steps: First, a binding step between the virus and the silica surface; second is to build a recognition layer which consists of silsequioxane that forms a cavity that is complementary to the virus; and third to remove the template. The mechanism by which VIPs work relies on their ability to mimic the biological recognition of natural systems. They found that this strategy worked well and exhibited a high binding affinity and selectivity for virus recognition which might be useful for other viruses as well [44].

Other viruses, such as Hepatitis A/B and Zika, have been detected using materials that sense for specific aptamers of the virus [44–51]. MIPs as biosensors have also been used to detect other viral proteins, such as bovine leukemia virus glycoprotein (gp51) [47], HIV type 1 glycoprotein [48] ,and the dengue virus protein (NS1) [49]. This same method can be used to detect the specific aptamers for SARS-CoV-2.

Such a method can be used for the detection of SARS-CoV-2 due to rapid mutations that are currently being observed by picking a specific virus aptamer and appropriate MIPs that contain acrylic groups as sensing materials. This approach can enhance the conductivity of the sensor and the acrylic groups could further be reinforced with conductive metal oxides.

Recently, Syritski and his team designed an MIP sensor to detect the SARS-CoV-2 nucleocapsid protein that protects the viral RNA as an antigen in a nasopharyngeal swab sample from a patient that had the disease [50]. The team designed a sensor on the chip that was endowed with a MIP that contained a nucleocapsid protein for SARS-CoV-2 for better selectivity and detection. The sensor was designed on a disposable Au-TFE chip with a MIP recognition site for the nucleocapsid virus protein, and that chip was connected to a tablet or cell phone for monitoring. The mechanism by which the sensor worked depended on the oxidation/reduction reaction probe and the charge transfer between Fe(CN)_6_]^3−^/[Fe(CN)_6_]^4−^. So, if the sample tested positive, the charge transfer would decrease, and the current would decrease. Additional researchers have designed an electrochemical gas sensor that consists of 4 layers and on the top of these layers, they used MI to detect the presence of SARS-CoV-2 in the air using the virus mutated spike proteins and Ebola. These layers included a silicon substrate, chromium layer for the conductivity of the previous layer, a graphene layer, and a Prussian-blue mixture. They found that this sensor can detect the presence of the virus in the air in less than 2 min, a potentially revolutionary technology using MIPs (unpublished data).

However, although there has been limited research in using MIP for improved COVID-19 diagnosis, there has been some promise. For example, Parisi and his coworkers synthesized MIP based on antibodies, used them as an alternative to traditional antibodies (which can be expensive, unavailable, a health concern, and overall difficult to work with) to recognize and bind to a specific sequence on the spike protein of SARS-CoV-2 to prevent its binding to the angiotensin-converting enzyme 2 (ACE2) which is the corresponding receptor on a host cell [51]. Three main approaches Parisi et al. considered when they designed the MIPs included the nature of the template, the specific sequence, and the binding between them [52]. Parisi et al. showed some promising preliminary results, but further studies are needed. Specifically, the MIP with the Coronavirus receptor binding protein showed binding affinity and detection by SDS-PAGE electrophoresis with good hemocompatibility. They synthesized a plastic antibody-based on MIPs that bound to the spike virus protein and blocked its function. Table 2 summarizes the use of MI in viral applications.

**Table 2:** Summary of MI use with viruses

Template virus	Imprinted on:	Applications
Small RNA plant viruses: *Turnip yellow mosaic virus* and *tomato bushy stunt virus*	Silica nanoparticles (SNPs)	Designing VIPs that have selective recognition to plant viruses [44]
Hepatitis A (HAV) and B viruses (HBV)	*N*-isopropylacrylamide (NIPAAm)	Detection and differentiation of HAV and HBV in human serum within 20 min [45]
Zika	Graphene	Detecting of the native Zika virus [46]
Bovine leukemia virus (BLV) glycoprotein 51(gp51)	Polypyrrole	Detection of BLV-gp51 [47]
HIV type 1 related protein (glycoprotein 41, pg41)	The surface of a quartz crystal microbalance (QCM) chip	Determining disease progression and the efficacy of drug intervention [48]
Dengue virus protein NS1 (pentadecapeptide; linear epitope of the virus)	The surface of a quartz crystal microbalance (QCM) chip	Detecting the Dengue virus protein [49]
Nucleocapsid protein of SARS-CoV-2	Au-TFE chip	Detecting for positive patients with the virus [50]
Mutated spike protein of SARS-CoV-2, SARS, Ebola	On the top of four sensor layers	Detecting the presence of the virus in air (unpublished data)
Spike protein of SARS-CoV-2	Plastic antibodies	Block the binding between the virus and ACE2 receptor [51]

#### Other MIPs for biomedical applications

MIPs can be used in many other applications in the medical field. Using MIPs as a drug delivery system (DDS) is a fascinating way to deliver a drug to its target and control its release, especially for a drug that is administered over a long period of time to enhance its effect, be metabolized and eliminated from the body [53, 54]. For example, polymeric nanoparticles that are designed from a mixture of methacrylic acid and N-hydroxyethyl acrylamide were used as an insulin-imprinted polymer to reduce insulin release in which insulin was used as a template [55]. That study showed an improvement in the absorption of insulin when it was taken orally due to MIP technology. These polymers are promising in numerous drug delivery and controlled release systems since they can selectively bind to many kinds of drugs, be resistant to a sudden change of pH or temperature, and protect the encapsulated drugs from proteolytic enzymes to allow them to reach the desired site [56–60] .Suksuwan and his team designed MIP nanoparticles to enhance the effectiveness of R-thalidomide which is an effective inhibitor of tumor necrosis factor-α to attack cells that resist a multitude of drugs [61]. Another example is using a magnetic nanocomposite of MIP with graphene oxide rather than using an adsorption mechanism of the drug (rivastigmine; a treatment for Alzheimer’s disease) within or on the polymers. The drug with MIP resulted in a pseudo first-order kinetic model that was highly biocompatible (84.4% cell viability) with a selectivity factor of 1.98 compared to non-imprinted polymeric samples [62]. Doxorubicin (DOX), which is an anticancer drug, is an example of a molecule that can be imprinted on the surface of mesoporous silica nanoparticles (MSNs) for improved DOX release [63]. That surface was stimulated by pH changes and glutathione. Studies have shown that 10.5 ± 0.2 wt% of Dox can be loaded into the MSNs with an efficacy of 70 ± 8%. Also, the results showed that a higher amount of the drug was released with a synergistic decomposition of the S–S bond at a low pH and in the presence of glutathione compared to physiological pH, which decreased its cytotoxicity compared to using DOX alone [63]. Specifically, the DOX alone killed 89% of cells at a concentration of 2.5 µg/ml but DOX imprinted polymers on the surface of the stable mesoporous silica nanoparticles (DOX@MSNs@MIP) killed 60% of cells at the same concentration. Importantly, the MIPs alone (MSN@NIP) did not show any toxicity on healthy cells (like TCA8113 cells) at concentrations from 0 to 100 µg/ml with cell viabilities near 100% [63]. Also, MIPs have played a critical role in antibacterial wound dressing applications. For example, Kurczewska and his co-workers encapsulated vancomycin which is an antibacterial drug with an alginate matrix for wound dressing applications. That matrix was then incorporated with an imprinted polymer and it was found that after incorporation with MIP, the drug was released at a slower rate compared with the polymer without alginate after 24 h (from 87 to 47%). The antibacterial activity was measured and showed a selectivity against killing certain bacteria strains and the release profile was controlled for diffusion and swelling [64]. All of these results indicated the advantages of using MIPs as an improved drug delivery system. Targeted drug delivery to kill infected cells selectively without causing any harmful effects on healthy cells is not easy. So, the overexpressed cells were targeted to bind to the surface of the drug carriers through the targeting moieties. Liu et al. further used the molecular imprinting procedure for targeting flexible peptide chains that undergo conformational changes, such as inducible epitope imprinting. They used MIP nanoparticles to adjust the peptides allowing them to specifically bind to the target protein. They found a stronger inhibitory effect using a xenograft mouse model after injection with these MIP compared with non–imprinted polymers [65]. The same group found a hidden epitope imprinting procedure where fibroblast growth factor-inducible 14 served as a target receptor that is overexpressed in solid tumor types. After the polymerization step, using the functional monomer, the peptides were converted from disordered to ordered structures. Then, the template was removed leaving empty cavities and the construction of these polymeric nanoparticles improved cellular uptake and permeability for targeting the tumor without changing the native conformation of the template peptide [66].

(S)-2-amino-3-(3,4-dihydroxyphenyl) propanoic acid (L-DOPA) is another example of a prodrug used to treat neurological disorders (such as Parkinson’s disease) but has limited stability in an aqueous solution [67]. L-DOPA alone has many limitations due to its polarity which does not allow it to cross the lipid barrier. Undoubtedly, there is an urgent need for nanocarriers to perform their function on a target site and protect it from degradation. So, by designing biodegradable polymers to encapsulate L-DOPA, its prolonged release would be enhanced [68]. Molecular imprinted technology can solve this problem and control and sustain its release for oral administration [69]. Specifically, when L-DOPA is imprinted and cross-linked with β-cyclodextrin and 1,1′-carbonyldiimidazole, the complex then allows for releasing L-DOPA in an aqueous solution [67]. MIPs are considered as the best method to help release L-DOPA in an aqueous solution due to the formation of non-covalent interactions unlike other polymers that covalently bind to drugs. MIP nanoparticles exhibit excellent clearance from the human body in urine and feces, which is consider one of their key advantages [70].

MIPs have also been used to deliver the prodrug 5′-Deoxy‐5‐fluorocytidine (DFCR) as a marker for tumor targeting (i.e., sialic acid) to a specific tumor without passing through the liver, which occurs with the drug alone and clearly reduces efficacy [71]. DFCR is an active form of 5-fluorouracil (FU) that inhibits thymidylate synthase which can lead to cell death. However, FU has many side effects [72]. DFCR was used as a prodrug in a recent study [71]. For this, with DFCR as a template, MIPs were synthesized by a boronate affinity pH-dependent and controllable oriented surface imprinting approach [73] by using sialic acid which is a saccharide overexpressed in cancer cells. These smart nanocarriers bound to the cancer cells since sialic acid and boronic acid have a high affinity to bind to tumor sites in a slightly acidic environment (pH 6.5–6.8) where cancer cells live. Then, the prodrug can be released to the tumor site and kill tumor cells [71]. Such a system helps to selectivity target the site of infection and release the drug in a more controlled manner and without passing through the liver than when using non-imprinted polymers.

**Fig. 6:**
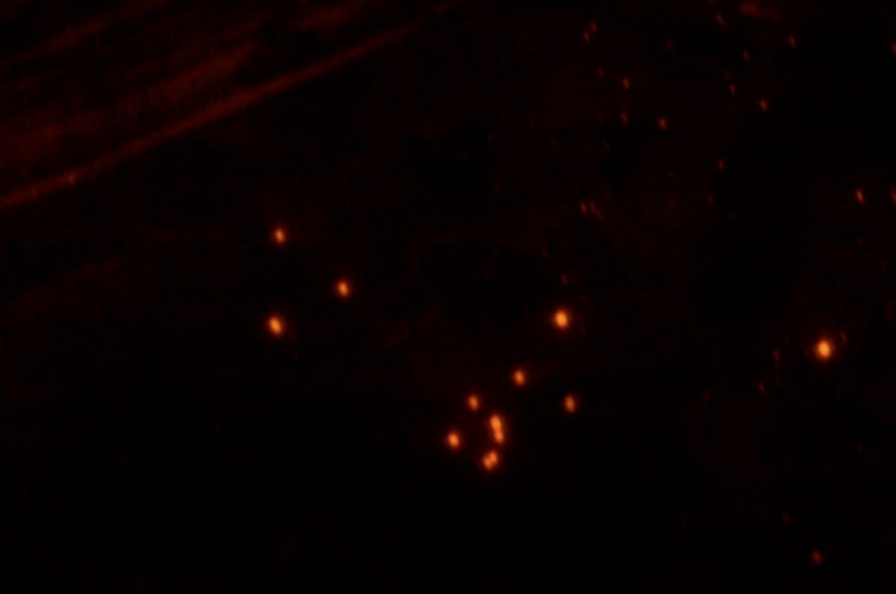
A fluorescence microscopy image of Sunitinib imprinted polymers with Rhodamine 6G at a magnification of ×40. Adapted from [75]

The MI process has also been used to imprint 5-fluorouracil on a magnetic fluorescent core or carrier [74]. Surprisingly, results showed that these magnetic carriers inhibited cancer cells more than normal cells, specifically, 25 times higher than when using the drug alone. From that result, researchers concluded that these magnetic nanocarriers could be effectively used to treat breast cancer [74]. Moreover, Parisi and his coworkers used MIPs as a theranostic system for the anticancer drug (Sunitinib) with Rhodamine 6G as a marker to control drug release and self-monitor cancer therapy due to the design of a fluorescent MIP with a high affinity to bind to Sunitinib [75]. So, these polymers not only act as a Sunitinib carrier, but also to monitor cancer cells which was the target of the study shown in Fig. 6. Using such a method would lead to the design of a theranostic system that can control drug release by monitoring it fluorescently. A theranostic system is an approach that is used to simultaneously diagnose a disease, monitoring drug response, and target a therapy [76]. Theranostic systems are very promising in cancer therapy and imaging and MIPs have significantly improved properties over conventional non-imprinted polymer systems for such applications. Table 3 summarizes some of the many biomedical applications of MI.

**Table 3:** Applications of MI in biomedical fields

MI with improved material functions	Polymers	Template	Applications
Insulin- imprinted polymer	Mixture of methacrylic acid and N-hydroxyethyl acrylamide	Insulin	Reducing insulin release especially when taking insulin orally [55]
DOX-imprinted polymer	Mesoporous silica nanoparticles	DOX	Improvement in efficacy and release of the drug [63]
L-DOPA-imprinted polymer	β-Cyclodextrin and 1,1′-carbonyldiimidazole	L-DOPA	Enhanced prolonged release of the drug with an excellent clearance profile [67]
DFCR-imprinted polymer	Boronic acid	DFCR	Helps in the control released of the drug and increased selectivity to the target site [71]
5-Fluorouracil- imprinted polymer	Magnetic fluorescent core	5-fluorouracil	Increased efficacy of the drug 25 times than the drug alone [77]
Sunitinib-imprinting polymer	Fluorescent MIP	Sunitinib	Monitoring the drug fluorescently [75]
B family vitamin-imprinting polymers	Hydrophilic multi-template MI biopolymers (mt-MIBP) using chitosan as a bio-substance	Riboflavin, nicotinamide, and pyridoxine	Separation and determination of these vitamins in orange juice [78]
Bovine hemoglobin- imprinting with a fluorescence sensor	Tricolor-emission sensor that was constructed with a mixture of blue, green, and red- emission MIPs	Bovine hemoglobin	Detection and visualize of bovine hemoglobin [79]

MIPs based on drug delivery systems have many advantages as you can see in the above section, such as enhanced stability, tailorability to select applications, and low cost, however, there are some challenges in using the drug delivery system for the commercial purposes. Some of these challenges are how the MIPs react with the surrounding environment, the efficacy of the drug loading, the selective recognition, the toxicity, biocompatibility of the polymers, and the balance between the pharmacokinetics and pharmacodynamics.

#### Self-assembled MI materials

Self-assembled materials form secondary bonds that self-assemble into controllable structures, thus, they are attractive for numerous MI approaches. The interactions between monomers and templates always depend on the arrangement (self-assembly) of the polymerized functional groups between them. These interactions are governed by covalent and noncovalent bonds. Most of these interactions are polar, ion-dipole, hydrogen bonding, or metal ion chelation. For example, methacrylic acid (MAA) is widely used as a monomer due to its non-covalent interaction and its favorable kinetic functions [80]. It has properties that self-assemble and act as a hydrogen donor or acceptor. In the MIP process, MAA with a template can polymerize and crosslink with ethylene glycol dimethacrylate and trimethylolpropane trimethacrylate. Then, the template is washed leaving behind empty sites complementary to the imprinted species becoming a memory recognition site [81].

The ratio of the monomer to the template in the MI process plays an important role to achieve a desirable self-assembled imprinted polymer. Too low of a ratio would cause insufficient functional groups for imprinted polymers; however, too high of a ratio would lead to steric hindrance and mismatching. Only the proper ratio of monomer to template leads to desirable polymers with high selectivity [82–84]; such a concept was monitored using UV spectroscopy [83]. For example, titration of a methacrylic acid (monomer) with a dipeptide as a template in a chloroform solution was monitored via UV absorption.

#### Assembled core-shell nanoparticles on MIPs

The core-shell on the surface of MIPs is an important area where the template is removed for the target to be re-bound [85–87]. This core could be made from magnetic nanoparticles [88], gold or silver nanoparticles [89], silica nanoparticles, or quantum dots [90, 91]. Each of these materials has properties that allow them to be suitable for specific medical applications. For example, silica is very stable under acidic conditions [92], and is biocompatible [93]. In order to enhance the properties of silica for selectively binding to a target, vinyl groups could be added [94]. Ma and his coworkers designed MIPs of 17 β-estradiol (E2) with silica nanoparticles (SiO_2_ @ E2-MIPs) and due to the core-shell material, the sorption capacity was five times higher than non-imprinted ones. Also, they found that the selectivity of the imprinted complex to estrogen was greater with faster kinetics [95]. These results can be used as an improved detector, monitor, or sensor for trace amounts of estrogen in duck feed. Estrogens can cause several health problems in women (like breast cancer) and have different effects on the endocrine system, so MI can help detect the presence of such hormones [96].

Core-shell MIPs could also be magnetic nanoparticles that include cobalt, iron, nickel, or alloys thereof [97–99]. Magnetic nanoparticles have many advantages for use in the biomedical field due to their high surface to volume ratios, effective binding to molecules, small size, and high magnetic susceptibility. So, when the magnetic nanoparticles are coated with MIPs, the analyte recognition selectivity in the matrix increases due to the targeted imprinting sites and from the high surface to volume ratio of the magnetic particles that provide greater surface polymerization [100, 101]. However, one of the main considerations when using these metals is their toxicity. Fe_3_O_4_ is one of the chemistries that exhibits lower cytotoxicity compared to others (such as cobalt and nickel) with higher biocompatibility rates [102]. Fe_3_O_4_ sizes ranging from 20 to 30 nm exhibited high viability on HeLa cells at a concentration of 10 µg/ml for 48 h where cytotoxicity started at a concentration of 100 µg/ml [103]. Magnetic nanoparticle core shells have been used for the recognition of bovine hemoglobin [100]. Semiconductor quantum dots (SQDs) are another example of nanoparticles used as core shells for MIPs due to their stability and high fluorescence intensity. The size of these dots ranges from 1 to 10 nm [104]. Another nanoparticle that has been used as a core shell include gold nanoparticles (Au) due to their nanoscale dimensions and large surface area [105]. Au nanoparticles as a core-shell can be used to support the synthesis of MI materials for the detection of cholic acid, which is a derivative from bile acids to determine its amount in blood and urine [105]. The concentration of bile acids in our body serves as a biomarker for some diseases like hepatitis, liver disease, and gallstones. Hence, monitoring such biomarkers using Au nano-sensors would be a useful diagnostic tool. There are other detection methods for bile acids in serum such as Ultra-High-Performance Liquid Chromatography-Mass Spectrometry (UHPLC) [106] and Liquid Chromatography (LC) with Ultraviolet (UV) detection [107], but all of these have limitations due to their cost, larger instrument size, and sophistication for use. Au nanoparticle core shells have also been used as an electrochemical sensor for the detection of dopamine in real urine and blood samples [108]. Dopamine is a neurotransmitter that exists in the central nervous system and brain, so any abnormal levels could lead to Parkinson’s disease, Schizophrenia, or other neurological disorders [109].

## Conclusions

Molecular imprinting has many promising biomedical applications especially those requiring selective binding to a target site. This approach has many advantages due to its cheap cost, ease of synthesis, and long-term stability. In this review, we covered the most recent promising applications of MIPs and how this approach can be used as a powerful tool to diagnose and monitor viruses, bacteria, or host cells attracting numerous researchers during the recent COVID-19 pandemic.

However, there are still some significant challenges when using this approach. Some of these challenges and limitations of using MIP are monomer selection, washing and removal methods, low binding capacity, undesirable side group interferences, poor efficacy, and reproducibility. One of these challenges is choosing the charged monomers because that could lead to strong electrostatic binding between a template and monomer where pH and ionic strength also affect binding. Researchers have solved this problem by using non-charged hydrophilic matrices. However, that is not the case with all MIPs since for the natural binding between the ligand and receptors, there would also be electrostatic interactions between them. For example, imprinting melittin, which is a polypeptide that contains 26 amino acids, can be achieved in an aqueous solution with N-isopropylacrylamide as a polymer and crosslinking with N,N′-methylene-bisacrylamide with monomers of acrylic acid N-t-butylacrylamide. These monomers bind to the template with electrostatic and hydrophobic interactions [110].

Also, template removal and rebinding need to be done with proper control. The kinetic measurements should be done with every batch to check for reproducibility [111]. Sometimes, it is a very strong interaction between monomers and the template or the detergent that is used in the removal step that could cause artifacts in the results. Lacking sensitivity and selectivity can be solved by using machine learning. Machine learning (ML) can predict imprinted polymer functionality before performing actual experiments to highlight the optimal interactions between the targeted template and the functional monomer interactions, thus, solving difficulties with the precise control of 3-D features. ML helps to accelerate the discovery of polymers with a great affinity in binding with the target with high thermal conductivity [112]. Other ways to improve the selectivity and sensitivity of MI is by using MI based on solid-phase extraction which is attractive today [111].

It is clear that MIP technology needs to be treated on a case-by-case basis, yet such efforts are well worth it, as covered here in this review, due to their powerful applications in different fields, especially medicine.

## Data Availability

Not applicable.

## Abbreviations

AB: 4'-Acetylbiphenyl
ACE2: Angiotensin-converting enzyme 2
COVID-2019: Coronavirus Disease of 2019
DDS: Drug delivery system
DFCR: 5′‐Deoxy‐5‐fluorocytidine
Dox: Doxorubicin
EGDMA: Ethylene glycol dimethacrylate
EGFR: Epidermal growth factor receptor
ELISA: Enzyme-linked immunosorbent assay
E. coli: *Escherichia coli*
FITC: Fluorescein isothiocyanate
FN: Fibronectin
FPBA: Fluorescent polymerization-based amplification
FU: 5-Fluorouracil
Fuc: Fucose
GlcA: Glucuronic acid
Gp41: Glycoprotein 41
Gp51: Glycoprotein 51
HAV: Hepatitis A virus
HBV: Hepatitis B virus
HepG-2: Human hepatoma carcinoma cells
LC: Liquid chromatography
L-DOPA: (S)-2-amino-3-(3,4-dihydroxyphenyl) propanoic acid
MAA: Methacrylic acid glacial
MAA: Methacrylic acid
Man: Mannose
MCF-10A: Mammary epithelial cells
MI: Molecular imprinting
MIPs: Molecular imprinted polymers
MSNs: Mesoporous silica nanoparticles
NANA: *N*-Acetylneuraminic acid
NIPAAm: N-Isopropylacrylamide
NIPAm: *N*-Isopropylacrylamide
NSE: Neuron-specific enolase
PBA: Phenylboronic acid
PFBT: Poly(fluorene-alt-benzothiadiazole)
RGD: Arginylglycylaspartic acid
RT-PCR: Reverse transcription-polymerase chain reaction
SA: Sialic acid
SAM: Self-assembled monolayer
SEM: Scanning electron microscopy
SNPs: Silica nanoparticles
SpA: *Staphylococcus aureus *
SQDs: Semiconductor quantum dots
TEOS: Tetraethoxysilane
UHPLC: Ultra-high-performance liquid chromatography
UV: Ultraviolet
VIPs: Virus imprinted polymers
QCM: Quartz crystal microbalance
